# A new species of *Lasiosmylus* from the Early Cretaceous, China clarifies its genus-group placement in Ithonidae (Neuroptera)

**DOI:** 10.3897/zookeys.636.10103

**Published:** 2016-11-24

**Authors:** Bingyu Zheng, Dong Ren, Yongjie Wang

**Affiliations:** 1 College of Life Sciences, Capital Normal University, 105 Xisanhuanbeilu, Haidian District, Beijing 100048, China

**Keywords:** Fossil, Huangbanjigou, ithonid genus-group, taxonomy, Yixian Formation

## Abstract

A new species, *Lasiosmylus longus* **sp. n.**, is described from the Early Cretaceous Yixian Formation of Huangbanjigou Village, Liaoning Province, China. Based on the characters of the new species and nine new specimens of *Lasiosmylus newi* Ren & Guo, 1996, the generic diagnosis of *Lasiosmylus* is emended and the taxonomic position of *Lasiosmylus* Ren & Guo, 1996 is re-evaluated, and *Lasiosmylus* should be assigned to the ithonid genus-group.

## Introduction

The genus *Lasiosmylus* Ren & Guo, 1996 was initially assigned to the subfamily Spilosmylinae in Osmylidae. Makarkin et al. (2012, 2014) then transferred it to Ithonidae *sensu lato*, but without discussing the relationship of the genus to other genera within the family. It is evident that the original assignment of *Lasiosmylus* to Osmylidae is questionable since the shared osmylid-like features discussed by the authors (i.e., absence of r1-rs crossvein, fewer crossveins throughout wing, and absence of gradate series) are not actual synapomorphies of Osmylidae. *Lasiosmylus* rather displays more typical ithonid-like characters, e.g., stout body, retracted head, distinctively narrowed costal space towards the pterostigma area and strongly recurrent humeral crossvein in forewing; undoubtedly, it is more suitable to attribute this genus to Ithonidae. At present, although it is widely accepted that Ithonidae comprise three lineages: ithonid genus-group (moth-lacewings), polystoechotid genus-group (giant lacewings), and rapismatid genus-group (montane lacewings), the interrelationships among these groups, especially for fossil taxa, are still not fully resolved (Winterton and Makarkin 2010, Makarkin et al. 2014, Zheng et al. 2016). As a result, most fossil taxa have been simply attributed to Ithonidae *sensu lato* without further systematic placement (Archibald and Makarkin 2006, Makarkin et al. 2014). Recently Zheng et al. (2016) proposed diagnostic features for the three lineages of Ithonidae, incorporating the extant and fossil taxa, which could form the basis for assignment of additional Ithonidae fossils.

In this study a distinctive new species of Ithonidae, *Lasiosmylus longus* sp. n., is described from the Early Cretaceous of Yixian Formation, China. Additionally, nine new fossil specimens assignable to *Lasiosmylus newi* Ren & Guo, 1996 were collected from the same locality, which allow us to re-evaluate the systematic position of the genus within Ithonidae. Based on this new information, the genus *Lasiosmylus* is attributed to the ithonid genus-group and the diagnostic characters of *Lasiosmylus* are amended.

## Materials and methods

**Figure 1.**
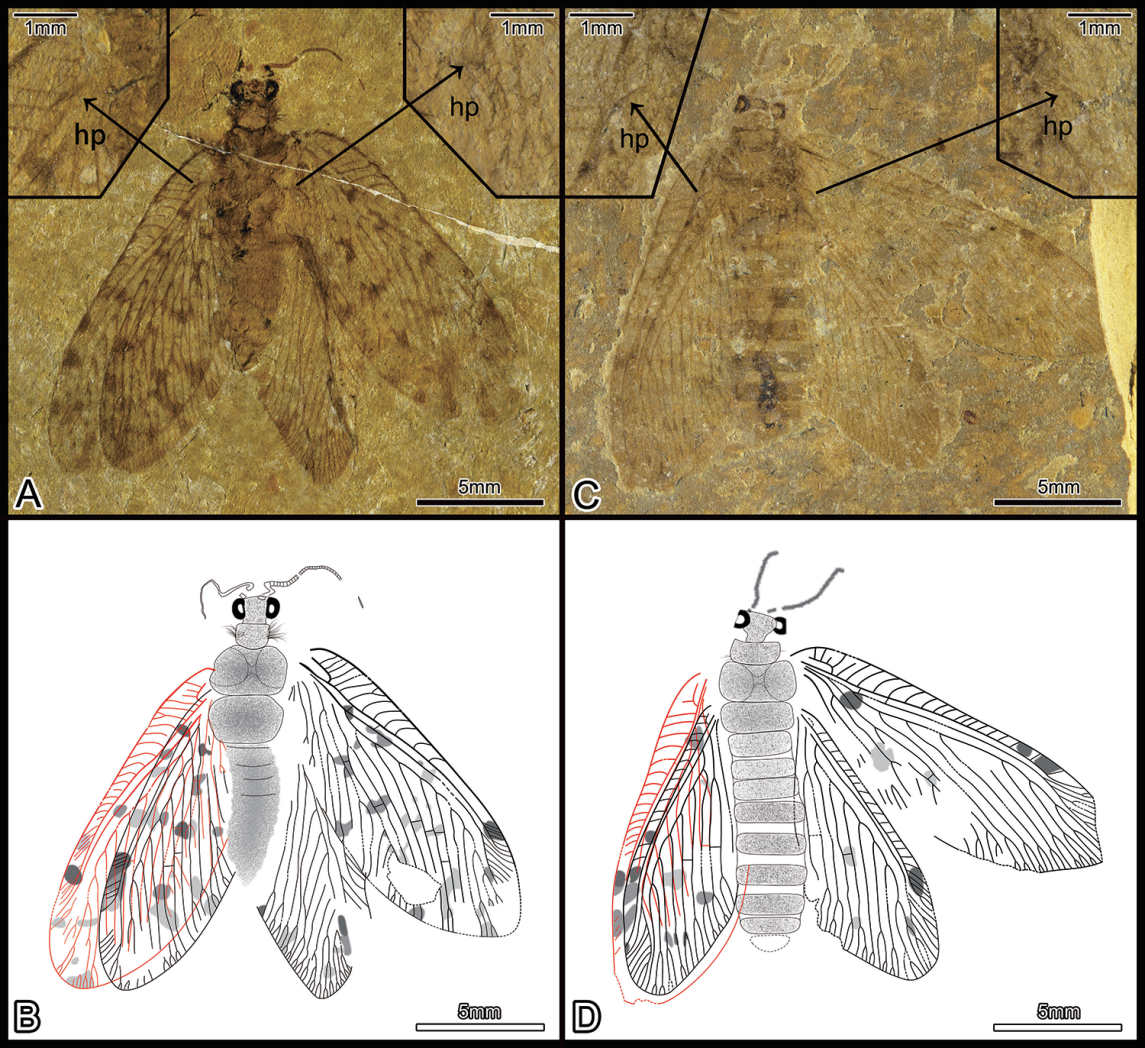
New materials of *Lasiosmylus newi*: CNU-NEU-LB2015001P/C, CNU-NEU-LB2015002. **A** composite photographs of habitus of part and counterpart (CNU-NEU-LB2015001P/C) hp, humeral plate (CNU-NEU-LB2015001C) **B** line drawing (CNU-NEU-LB2015001C) **C** habitus photograph, hp, humeral plate (CNU-NEU-LB2015002) **D** line drawing (CNU-NEU-LB2015002). Scale bars: 5 mm (**A–D**).

This study is based on ten specimens, which are deposited in the Key Lab of Insect Evolution and Environmental Change, Capital Normal University, Beijing, China. Draft drawings were produced using LEICA MZ75 dissecting microscope equipped with a drawing tube. Drawings were finalized using Adobe Illustrator CC. Photographs were taken by Leica Digital Camera DFC500 (Figs 1A, C) and Nikon Digital Camera SMZ25 (Fig. 3A), and produced with Adobe Photoshop CC. Additionally, the part of one specimen (CNU-NEU-LB2015001P) was fragmented and glued loosely during collecting, the counterpart of the specimen (CNU-NEU-LB2015001C) is complete. A composite photograph of the part and counterpart is shown on Fig. 1A, which is the combination of two photos from both parts of the specimen in dry condition. The technique of the composite photograph in this study follows that of Béthoux (2015).

The terminology of venation in general follows Barnard (1981), except the terminology of humeral plate follows Oswald (1993):

Sc
 Subcosta 


R1
 first branch of Radius  (**R**)

Rs
 Radial sector 


MA
 anterior branches of Media  (**M**)

MP
 posterior branches of Media 


CuA
 anterior Cubitus  (**Cu**)

CuP
 posterior Cubitus 


1A–3A
 Anal veins 


hp
 humeral plate 


hv
 humeral veinlet 


## Systematic paleontology

### Order Neuroptera Linnaeus, 1758 Family Ithonidae Newman, 1853 *sensu* Winterton & Makarkin, 2010

#### Genus Lasiosmylus

Taxon classificationAnimaliaNeuropteraIthonidae

Ren & Guo, 1996

##### Type species.

*Lasiosmylus newi* Ren & Guo, 1996.

##### Species included.

*Lasiosmylus newi* Ren & Guo, 1996, *Lasiosmylus longus* sp. n.

##### Amended diagnosis.

Body stout (ca. 11–17 mm long), covered with dense setae; head hypognathous, protruding from pronotum partly; antenna filiform (ca. 2–5 mm, incompletely preserved); compound eye large, ocelli absent; thorax robust, long setae concentrated on pronotum. Forewing ca. 12–23 mm long, 5–8 mm wide, membranous area with many fuscous spots; humeral plate distinct; dense setae along the veins, especially on the wing margin; trichosors and nygmata undetectable; costal space dilated basally and narrowed distally; humeral veinlet recurrent, with several simple branches; costal cross-veins simple, moderately curved distally in the apical half of the costal space; Sc and R1 separate distally, entering the margin before the wing apex; one or two sc-r1 crossveins; R1 with four to eleven pectinate branches distally; the origin of Rs distant from the wing base, with seven to thirteen branches regularly arranged; relatively few crossveins present in radial area; MA simple, dichotomously branched terminally; MP first fork distant from wing base. Hind wing ca. 11–18 mm long, 4–8 mm wide, partly preserved, venation similar to forewing except for the following characters: costal space narrow, only slightly expand in proximal portion.

##### Remarks.

*Lasiosmylus* shows a superficial similarity with osmylids, sharing plesiomorphic features such as the fork of MP in forewing usually between the separation of MA and first Rs branch, sometimes opposite the separation of MA; wings not falcate, with few crossveins (Ren and Guo 1996). However, all these characters do not well support the assignment of *Lasiosmylus* to Spilosmylinae, or Osmylidae in general because they also frequently occur in other families (e.g., Ithonidae, Berothidae, some Mantispidae). The subsequent transfer to Ithonidae by Makarkin et al. (2012, 2014) seems reasonable; moreover, recently it was classified further as belonging to the polystoechotid genus-group by Zheng et al. (2016).

**Figure 2.**
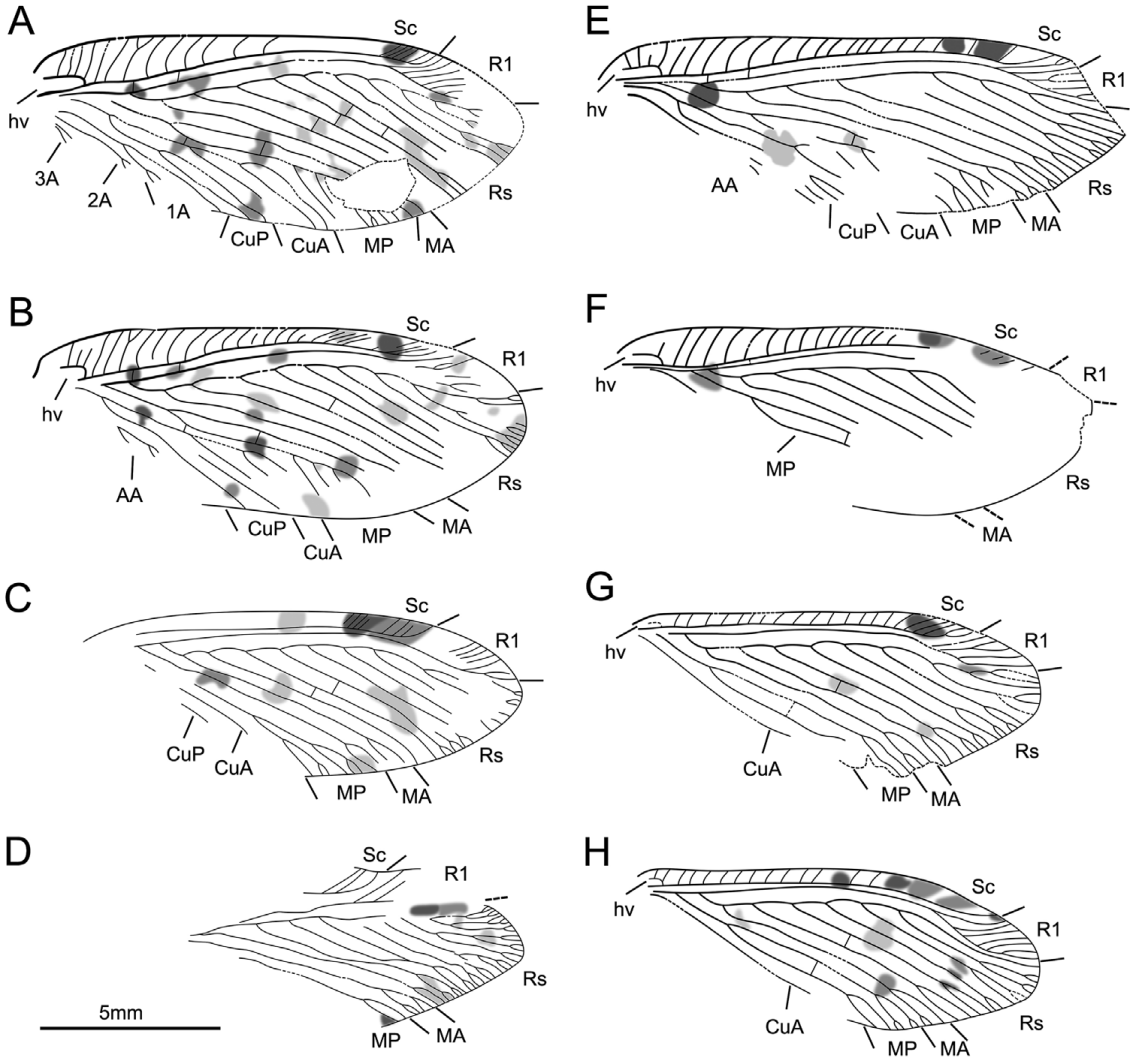
New materials of *Lasiosmylus newi*. Line drawings of CNU-NEU-LB2015001P/C, **A** left forewing **B** right forewing **C** right hind wing **D** left hind wing. Line drawings of CNU-NEU-LB2015002 **E** left forewing **F** right forewing **G** left hind wing **H** right hind wing. Scale bars: 5 mm (**A–H**).

Herein, nine new-collected specimens are examined in this study. All these specimens are placed in *Lasiosmylus* based on the following characters: numerous dispersed spots on the forewing, simple costal crossveins, two subcostal crossveins, Rs less than ten branches (about six to eight branches), MP distant from the wing base and beyond MA fork, MP1 and MP2 simple, one mp1-mp2 crossvein, CuA dichotomously branched distally (in particular, obs. CNU-NEU-LB2015001P/C and CNU-NEU-LB2015002, see Figs 1, 2; and Ren and Guo 1996: fig. 5, fig. 10, pl. 3, fig. 11, pl. 2). Noticeably, during checking the specimens, we found some variable characters that are distinctly different from the type specimen, e.g., humeral veinlet and separated Sc and R1. A recurrent humeral veinlet is considered as a synapomorphy for Ithonidae (Yang et al. 2012, Makarkin et al. 2013, Zheng et al. 2016). However, this character is absent in the line drawing of *Lasiosmylus newi* (Ren and Guo 1996: fig. 5), although some trace of recurrent humeral veinlet can be detected in the photograph of *Lasiosmylus* (Ren and Guo 1996: fig. 11, pl. 4). Regretfully, the holotype of *Lasiosmylus newi* was not available for examination during this study (possibly lost). However, it is reasonable to assume now that the recurrent humeral veinlet occurs in *Lasiosmylus newi* according to these new specimens.

In addition, the distally separated Sc and R1 were regarded as a synapomorphic character of Ithonidae (Zheng et al. 2016). In the original illustration of *Lasiosmylus newi*, Sc and R1 were drawn with fused termination. Unfortunately, the photograph of *Lasiosmylus newi* is too obscure for us to discern the condition of Sc and R1 (Ren and Guo 1996: fig. 10, pl. 3, fig. 11, pl. 2). In extant members of the polystoechotid genus-group Sc and R1 are closely approximated but are actually not fused, e.g., *Fontecilla* Navás, 1931, *Platystoechotes* Carpenter, 1940, *Polystoechotes* Burmeister, 1839 (see Winterton and Makarkin 2010). While this character was not fully investigated in the fossil lineages, most fossil polystoechotid genera were illustrated with the fused Sc and R1.

During the examination of the new materials, it is clear that all specimens assigned to *Lasiosmylus* (Figs 1, 2) show a separate Sc and R1. Furthermore, nine specimens (CNU-NEU-LB2015001P/C, CNU-NEU-LB2015002, CNU-NEU-LB2016001P/C, CNU-NEU-LB2016002, CNU-NEU-LB2016003, CNU-NEU-LB2016004, CNU-NEU-LB2016005, CNU-NEU-LB2016006, CNUNEU-LB2016007) exhibit the typically venation with *Lasiosmylus newi* with exception for the incompatible conditions of Sc and R1. These nine specimens are considered to be *Lasiosmylus newi*.

It is concluded here that the genus *Lasiosmylus* most commonly has the separated Sc and R1 that is consistent with other moth lacewings. The exception of Sc and R1 in the holotype of *Lasiosmylus newi* possibly represents a particularly individual variation, inaccuracy in line drawing or obscurity in the specimen. Based on this we consider *Lasiosmylus* is unquestionably assigned to the ithonid genus-group by the following combination of characters: robust and hairy body, retracted head under pronotum, costal space dilated basally and narrowed disproportionately distally, separated Sc and R1 reaching the anterior margin straightly before the wing apex, MP first fork distant from the wing base and beyond the divergence of MA.

#### Lasiosmylus longus sp. n.

Taxon classificationAnimaliaNeuropteraIthonidae

http://zoobank.org/66865D0B-21B0-42C4-BDD5-98CE8AE31A2D

Figs 3 , 4

##### Material.

Holotype, CNU-NEU-LB2015003, a partly preserved specimen. Body barely preserved, but four overlapping, sub-complete wings, partially folded, with visible features.

##### Diagnosis.

Humeral veinlet recurrent, with a few branches; numerous markings present on the forewing; a distinct oblique stripe parallel to the outer margin; costal crossveins simple; one basal subcostal crossvein; Sc and R1 separate distally, Sc terminating in costal margin 2/3 length of wing; R1 with numerous anteriorly directed branches; Rs with more than ten branches; MP fork level with origin of MA; CuA pectinately branched, CuP with three distal branches.

##### Description.

**Figure 3.**
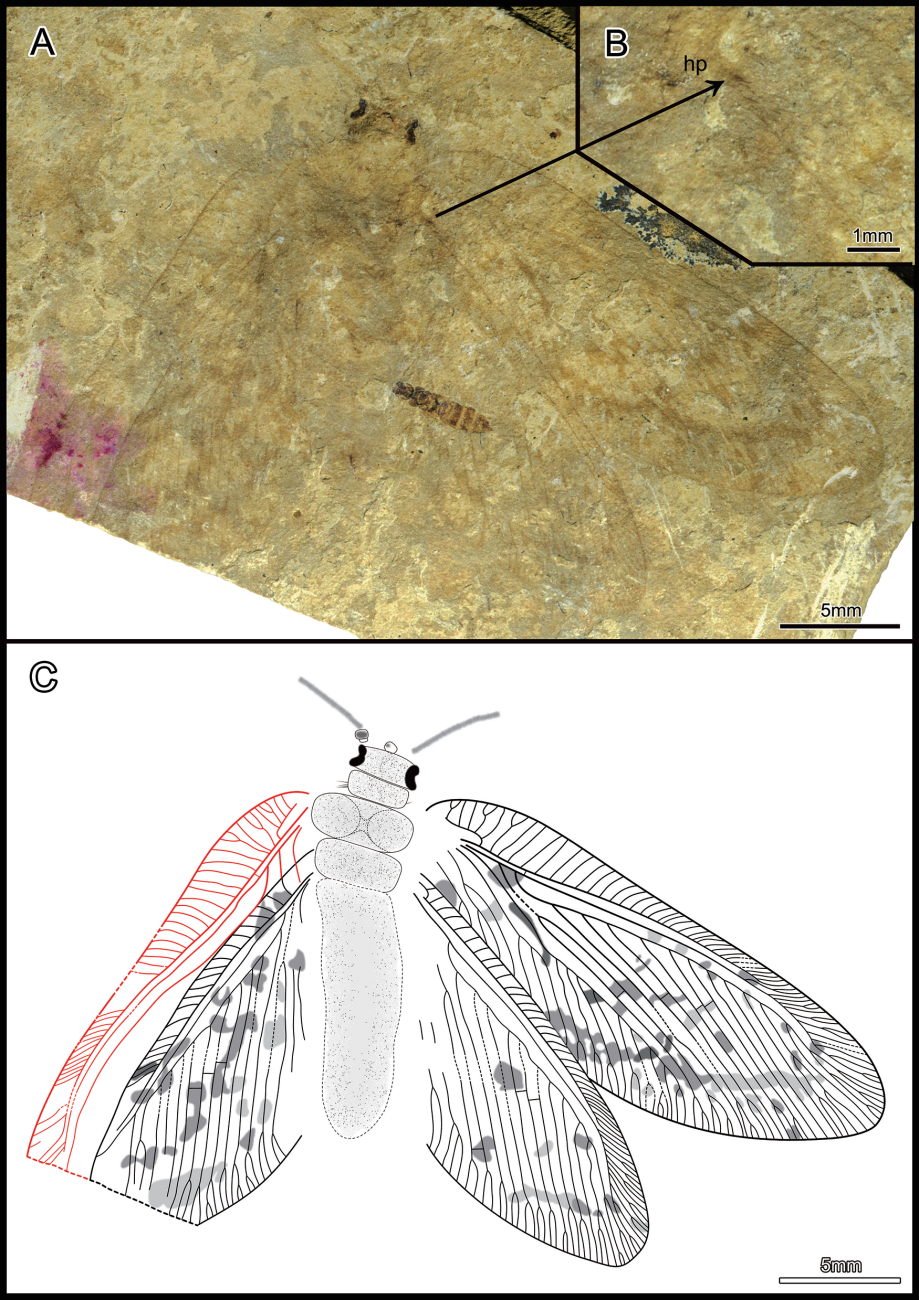
*Lasiosmylus longus* sp. n. (holotype CNU-NEU-LB2015003). **A** habitus photograph **B** hp, humeral plate (left hindwing) **C** line drawing. Scale bars: 5 mm (**A, C**), 1 mm (**B**).

**Figure 4.**
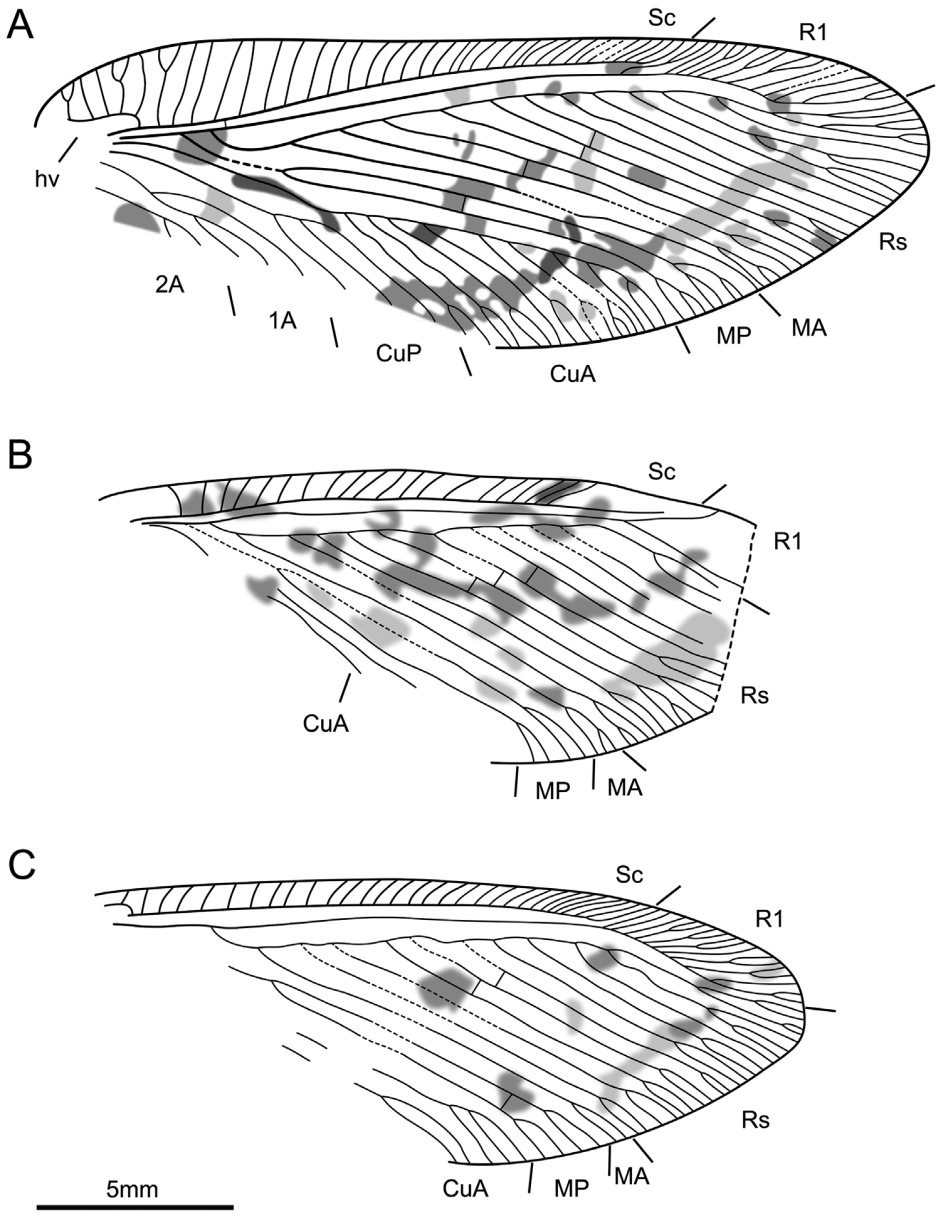
*Lasiosmylus longus* sp. n. (holotype CNU-NEU-LB2015003), line drawings. **A** right forewing **B** left hind wing **C** right hind wing. Scale bars: 5 mm (**A–C**).

*Body*: ca. 16.3 mm long; head hypognathous, retracted into pronotum partly; antenna filiform (ca. 4.0 mm) and incompletely preserved; compound eye large, ocelli absent; pronotum quadrate, numerous long setae concentrated laterally; mesonotum and metanotum stout; abdomen and legs indiscernible. *Fore wing*: ca. 22.7 mm long, 7.9 mm wide; slender and membranous with numerous fuscous spots; humeral plate discernible (Fig. 3A); veins covered by dense setae, particular along wing margin; trichosors and nygmata undetectable; costal space broad basally (maximum width = 2.1 mm), narrowed distally; recurrent humeral veinlet with several branches; costal crossveins simple and with the occasional distal dichotomous forks, densely arranged distally; Sc and R1 separated distally; one subcostal crossvein close to the origin of Rs; R1 with many pectinately branches distally, entering the anterior margin; Rs branches regularly arranged with about thirteen branches; few crossveins present between branches of Rs; MA simple; MP first fork distant from wing base, close to the MA divergence from Rs; one mp1-mp2 crossvein detected; CuA branched near the middle of wing, with ten pectinate branches; CuP with three simple branches; anal veins partly preserved, 1A with three branches and forked proximally, 2A proximally forked. *Hind wing*: ca. 18.0 mm long, 7.3 mm wide, partly preserved, venation similar to forewing except costal space narrow; cubitus veins and anal veins not well preserved (Figs 3, 4).

##### Etymology.

The species name is from the Latin ‘*longus*’, referring to the slender wing of this moth lacewing.

##### Type locality.

Huangbanjigou Village, Beipiao City, Liaoning Province, China.

##### Type horizon.

Yixian Formation, Barremian-early Aptian (129.7–122.1 Ma), Early Cretaceous.

##### Remarks.

*Lasiosmylus longus* sp. n. can be distinguished from *Lasiosmylus newi* by the distinct oblique stripe close to the outer margin, multiple Rs branches, and pectinate CuA branches.

## Supplementary Material

XML Treatment for Lasiosmylus

XML Treatment for Lasiosmylus longus
